# Gut Microbiota-Derived Metabolites in the Development of Diseases

**DOI:** 10.1155/2021/6658674

**Published:** 2021-01-08

**Authors:** Guangyu Shen, Jing Wu, Bang-Ce Ye, Nan Qi

**Affiliations:** Institute of Engineering Biology and Health, Collaborative Innovation Center of Yangtze River Delta Region Green Pharmaceuticals, College of Pharmaceutical Sciences, Zhejiang University of Technology, Hangzhou 310014, Zhejiang, China

## Abstract

Gut microbiota is increasingly recognized as a metabolic organ essential for human health. Compelling evidences show a variety set of links between diets and gut microbial homeostasis. Changes in gut microbial flora would probably contribute to the development of certain diseases such as diabetes, heart disease, allergy, and psychiatric diseases. In addition to the composition of gut microbiota, the metabolites derived from gut microbiota have emerged as a pivotal regulator in diseases development. Since high-fat and high-protein diets substantially affect the gut microbial ecology and human health, the current review summarizes the gut microbiota-derived metabolites such as short-chain fatty acids (SCFAs), amino acids, and their derivatives and highlights the mechanisms underlying the host responses to these bioactive substances.

## 1. Introduction

Intestine is a complex ecosystem harboring a diversity of microbial community known as the gut microbiota. Gut microbiota has recently emerged as a virtual endocrine organ producing multiple compounds, which maintains the homeostasis and influences the function of the human body. The gut microbiota community is predominantly composed of two phyla: Firmicutes and Bacteroidetes [1–4]. Emerging data show that an aberrant gut microbiota composition is associated with several diseases, such as metabolic disorders and inflammatory bowel disorder (IBD) [5]. Prebiotic feeding (e.g., with inulin-type fructans and some polyphenols) strongly increases the presence of *A. muciniphila* and improves metabolic disorders [6]. Conversely, some studies in mice have reported an increased abundance of *A. muciniphila* on the ingestion of a high-fat high-sucrose diet [7]. The host diets are believed to regulate the composition of gut microbiota and microbiota-derived metabolites, which causes a crosstalk between the host and its microbiome. A growing body of research has focused on the microbially produced metabolites such as short-chain fatty acids (SCFAs), amino acids, and their derivatives cometabolized by the host [8]. Implications from the diet–microbiota–host interactions highlight the therapeutic potential for preventing and treating certain diseases. Koh et al. identifies imidazole propionate as a microbially produced histidine-derived metabolite that is present at higher concentrations in subjects with type 2 diabetes [9]. In this review, we will describe the microbial origin of several key metabolites produced from diets and their remarkable effects on host physiology.

## 2. Synthesis of Short-Chain Fatty Acids

Dietary fibers but also proteins and peptides, which escape digestion from host enzymes in the upper gut, are metabolized by the microbiota in the cecum and colon [10]. Short-chain fatty acids are the metabolites of dietary fibers metabolized by intestinal microorganisms [11]. Protein fermentation can also contribute to the SCFAs pool but mostly gives rise to branched-chain fatty acids such as isobutyrate, 2-methylbutyrate, and isovalerate exclusively originating from branched-chain amino acids valine, isoleucine, and leucine [12]. However, branched-chain amino acids (BCAAs) have been proposed as potentially harmful microbially modulated metabolites [13–15]. The acetate (C2), propionate (C3), and butyrate (C4) are the most abundant (≥95%) SCFAs, which are saturated aliphatic organic acids that consist of one to six carbons. Acetate, propionate, and butyrate are present in an approximate molar ratio of 3 : 1 : 1 in the colon and stool [15, 16]. In the cecum and large intestine, 95% of the produced SCFAs are rapidly absorbed by the colonocytes, while the remaining 5% is secreted in the feces. The SCFAs are not distributed evenly, which means they are decreased from proximal colon to distal colon [17]. Changing the distribution of intestinal flora and thus the distribution of metabolites may be of a great effect in the treatment of diseases because there is a concentration threshold for acetate's different impacts on the host.

**Figure 1:**
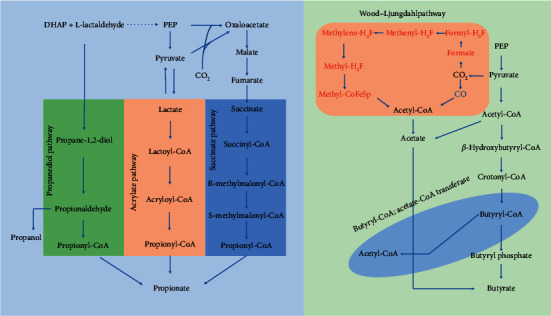
Pathways for biosynthesis of propionic acid, acetic acid, and butyric acid in microorganism.

Conceptually, the simplest way to synthesize an organic molecule is to construct one carbon at a time. The biochemical events that underlie the condensation of two one-carbon units to form the two-carbon compound, acetate, have intrigued chemists, biochemists, and microbiologists for many decades [18]. Gut microbiota produce acetate from (1) the pyruvate pathway which can produce acetyl-CoA as the precursor for acetate (2) and the Wood–Ljungdahl pathway which is composed of two branches: (1) the C1-body branch (also known as eastern branch) via reduction of CO_2_ to formate and (2) the carbon monoxide branch (the western branch) via reduction of CO_2_ to CO [19]. End product of this pathway is acetyl-CoA which is formed by formate, CO, and the extra methyl group. Another major SCFA butyrate has a particularly important role as the preferred energy source for the colonic epithelium and a proposed role in providing protection against colon cancer and colitis [20]. The two molecular of acetyl-CoA are converted to butyryl-CoA; then, the butyryl-CoA is turned into butyrate by some gut microbe with phosphotransbutyrylase and butyrate kinase. Interestingly, some microbes possess an enzyme called butyryl-CoA: acetate-CoA transferase, which transforms acetate and butyryl-CoA into acetyl-CoA and butyrate. There is a connection between acetate and butyrate distinctly, which suggests the complexity of the relationship between metabolites and leads us to think the significance of this metabolite transformation for the survival of bacteria. The significance may even play an important role in disease development. Propionate is produced in the human large intestine by microbial fermentation and may help maintain human health that includes antilipogenic, serum cholesterol levels lowering, anti-inflammatory, and anticarcinogenic functions [21–23]. There are three major microbially produced ways [24]: (1) acrylate pathway, (2) propanediol pathway, and (3) succinate pathway, which involves three genes: *lcdA* (encoding lactoyl-CoA dehydratase), *pduP* (encoding propionaldehyde dehydrogenase), and *mmdA* (encoding methylmalonyl-CoA decarboxylase), respectively Figure 1. Of note, diet fibers are not the only source for SCFAs. Analysis of metagenome data also suggested that butyrate can be synthesized from proteins via the lysine pathway [25]. Consequently, there are bacteria with different functions in the intestinal tract, and they perform their own duties. Some of them provide specialized supports for other functional bacteria or intestinal cells such as producing nutrients such as SCFAs. These kinds of bacteria are just like producers in the ecosystem. And these bacteria are promising to be migrated objects for fecal microbiota transplantation (FMT).

### 2.1. Regulation of Glucose/Energy Metabolism by SCFAs

The regulation of glucose metabolism by SCFAs is determined by multiple mechanisms. A recent study suggested that acetate, in the form of neutralized AcOH, activated AMPK (5′-AMP-activated protein kinase) in rat hepatocytes [26]. Activation of the hepatic AMPK pathway decreased gene expression of the gluconeogenic enzymes glucose-6-phosphatase (G6Pase) and phosphoenolpyruvate carboxykinase (PEPCK). AMPK mediates glucose uptake and free fatty acid oxidation in skeletal muscle and inhibits gluconeogenesis, glycolysis, lipogenesis, and cholesterol formation in the liver. Propionate, itself a substrate of IGN (intestinal gluconeogenesis), activates IGN gene expression via a gut-brain neural circuit involving the fatty acid receptor FFAR3 [27]. Though there is a plausible contradiction that SCFAs play opposite roles in gluconeogenesis. SCFAs have beneficial effects on glucose and energy homeostasis beyond question. The FFAR3 reporter is strongly expressed in the main, large population of enteroendocrine cells throughout the GI tract (gastrointestinal tract) but surprisingly also in neurons of both submucosal and myenteric ganglia. In contrast, the FFAR2 is expressed only in a subpopulation of the enteroendocrine cells but very strongly in a large population of leukocytes in the lamina propria throughout the small intestine [28]. SCFAs can activate FFAR2/3 (GPCR43/41) in colon cells to secrete PYY (peptide YY) or GLP-1 (glucagon-like peptide-1) into plasma. It is proved that GLP-1 can promote the secretion of insulin and inhibit the secretion of glucagon. The PYY can improve glucose uptake and utilization of periphery tissues [29]. What is more, another G protein-coupled receptor TGR5 responsive to bile acids can fine-tune energy homeostasis as a part of the BA–TGR5–cAMP–D2 signaling pathway [30] that can be targeted to improve metabolic control. The PYY or GLP-1 secreted by the intestinal cells mediate the nucleus tractus solitarius (NTS) in the brain via the vagus nerve and the circulatory system. Then, the signal is transmitted to ARC (arcuate nucleus) in the hypothalamus to enhance the satiety [31]. What is more, SCFAs existing in human cerebrospinal fluid work as an important energy source for glial cells and initiate peripheral eﬀects such as enhanced leptin production by adipose tissue or diminished ghrelin production in the stomach [32]. Further investigations are needed to elucidate the complicated gut-microbiota-brain axis and the potential of gut-microbiota-targeted strategies, such as dietary interventions and faecal microbiota transplantation (FMT) that help patients to live a healthy weight throughout life. Some researchers have improved pseudomembranous colitis by faecal microbiota transplantation [33].

### 2.2. Relationship between Cancer and SCFAs

Each nucleosome contains a nucleosome core, composed of an octameric complex of the core histone proteins, which forms a spool to wrap 145–147 bp of DNA [34]. The nucleosome core with about 165 bp of DNA together with the linker histone is called the chromatosome. The level of histone acetylation can influence the DNA replication; thereby, it determines cell proliferation in some way. There are two enzymes called histone acetyltransferase (HAT) and histone deacetylase (HDAC), which promote gene transcription and inhibit gene transcription, respectively. When losing the steady state of some gene expressions regulated by these two enzymes, the cells get high-risk differentiating into cancer cells. The SCFAs are one of the well-known HDAC inhibitors (HDACi) which have been used for cancer therapy. Among these SCFAs, the butyrate is the most pop and promising modulator of cancer and immune homeostasis. The butyrate is the primary energy source for colonocytes by transporting into colonocytes, entering the mitochondria, and undergoing *β*-oxidation to acetyl-CoA. Consequently, the acetyl-CoA enters the TCA cycle resulting in the reduction of NAD + to NADH, which enters the electron transport chain culminating in ATP production with CO_2_ as a byproduct. Thus, butyrate has been shown to stimulate cell proliferation in a low concentration as a HAT activator. However, the nutritional function of butyrate is also important that it is proven to have a regulation on autophagy when the colonocytes are in an energy-deprived state via AMPK and p27 [35]. Naturally, butyrate also exerts antiproliferative and anticancer effects when tumor cell lines are exposed to it in vitro, primarily through HDAC inhibition. The Warburg effect (aerobic glycolysis) indicates that the cancerous colonocytes prefer glucose rather than butyrate as the energy substrate. For this reason, butyrate could be accumulated to a high concentration where it can protect against colorectal cancer as HDACi. It is interesting that butyrate has chance to play a HDACi role just because of the “strange food preferences” of cancerous colonocytes. The strange food preferences mean that cancerous colonocytes prefer glucose as the energy source rather than butyrate. In conclusion, the butyrate has totally different functions in different situations. A recent study showed that gut microbial production of butyrate stimulated polyp formation in a genetic mouse model of colorectal cancer (*Apc*^*Min/+*^*Msh2*^*−/−*^) [36]. The keypoint is that the polyp formation is considered as the marker of colorectal cancer. Thereby, the accurate relationship between butyrate and host disease development is not totally rigorously studied yet. One day, using butyrate as clinical application should take more individual differences and situations into account.

### 2.3. Relationship between Short-Chain Fatty Acids and Inflammation

SCFAs can modulate the progression of inflammatory diseases either by inhibiting histone acetylase (HDAC) activity, and thereby affecting gene transcription, or through the activation of metabolite-sensing G-protein coupled receptors (GPCRs) such as GPR43. Numerous works have proven that the SCFAs are related to decreasing of cytokines such as IL-6 and IL-8 in human macrophages [37] and TNF*α* in peripheral blood mononuclear cells (PMBCs) [38]. These inflammation-related phenomena are bound up with the HDAC inhibition role of SCFAs. There are general two steps for maturation of cytokines: proinflammatory and inflammatory. Thus, the anti-inflammatory effect of SCFAs could mediate inflammation by inhibiting gene which encodes cytokines or the mediator involved in production of mature inflammatory cytokines. Butyrate and propionate are found inhibiting the NF-kB pathway which is activated to release inflammatory cytokines. Thus, the inhibition role of SCFAs to HDAC may work through modulating NF-kB activity via controlling DNA transcription [38]. Macrophage is one kind of white blood cells, derived from monocytes. Once stimulated, macrophages rapidly produce a large number of TNF-*α*, IL-1*β*, IL-6, NO, and arachidonic acid derivatives [39]. Numerous studies have established a role for butyrate that it inhibits macrophage migration mediated by LPS via reducing the transcriptional activity of Src (a nonreceptor tyrosine kinase) [40]. Regulatory T  cells (Tregs) are considered sensitive to HDAC inhibition, which may be resulted by increased Foxp3 (forkhead box P3) induction through acetylation at *FoxP3* locus [41, 42]. Foxp3 is a transcription factor necessary for Treg development and function. Thus, SCFAs could mediate proliferative and functional capabilities of Tregs via *Foxp3*. The butyrate could slightly diminish the proliferation of Tregs but enhance the inhibitory ability on T cell proliferation mediated by CTLA-4 [43]. SCFAs are also important for regulating effector T cells such as CD4^+^ and CD8^+^ T cells particularly in regards to increased function and differentiation.

The integrity of epithelial is important for intestinal homeostasis because a leaky intestinal mucosal barrier allowed more intestinal microbial metabolites appear at where they should not be, which may initiate lots of unnecessary inflammation. SCFAs showed increasing anti-inflammatory IL-18 secretion by intestinal epithelial cells (IECs) [44], and IL-18 is a cytokine promoting gut epithelial integrity [45]. Butyrate-stimulated signaling of GPR109A could induce differentiation of Tregs and IL-10-producing T cells [46]. While GPR43 is activated by all three SCFAs, GPR109A is activated only by butyrate [47, 48]. Acetate was shown to promote the release of ROS (reactive oxygen species) when added on mouse neutrophils by activating GPR43 [49]. ROS is thought to upregulate or inhibit inflammation in a concentration-dependent manner. Therefore, the specific functions of SCFAs on controlling inflammation are supposed to be discussed in multiple views. The GPR43 also activates the NLRP3 inflammasome, which is critical for intestinal homeostasis. There are two stages of NLRP3 inflammasome including priming phase and signal activation [50]. The GPR43 activated by acetate initiate the hyperpolarization due to K+ efflux or successive to Ca^2+^ mobilization happening, which activates the NLRP3 inflammasome [44]. This is consistent with the downstream increasing IL-18. This beneficial role on epithelial integrity was confirmed in a model of dextran sulphate sodium- (DSS-) induced colitis in vivo in which the protective role of dietary fiber was mediated through NLRP3 activation in the epithelial compartment following GPCR activation [44]. In general, the SCFAs are multifunctional gut microbial metabolites that are of benefit to the host. The applications of it on different diseases should be more cautious because of its multifunction, which could initiate other chain reactions that we do not hope.

### 2.4. Aromatic Amino Acid Metabolites

The human digestive system will hydrolyze the proteins from all kinds of food into amino acids with the help of various proteases. A growing body of knowledge [51, 52] is accumulating by metabolomics that points the gut microbiota is also a mediator of the host health status via amino acids metabolism. The aromatic amino acid is called essential amino acid including tyrosine, tryptophan, and phenylalanine, which cannot be synthesized in vivo. Histidine is also an aromatic amino acid because of its imidazole ring. Microbially produced imidazole propionate from histidine is proven to impair insulin signaling through mTORC1 [9]. Thus, interactions among the gut microbiota, diet, and the host potentially contribute to the development of metabolic diseases and deserve more research.

### 2.5. Tryptophan Metabolites

Since the tryptophan (Trp) is not produced by animal cells, human rely on exogenous, mostly dietary intake. Tryptophan and its derivatives, bioactive small molecules, originate from nutrition- and environmental-related sources or are endogenously produced and modulated by the host and its microbiota. The three currently most studied pathways of tryptophan metabolism involved in host-microbiota interactions are as follows [51]: (1) the direct transformation of tryptophan into several molecules, including ligands of the aryl hydrocarbon receptor (AhR), by the gut microbiota; (2) the kynurenine pathway (KP) in both immune and epithelial cells via indoleamine 2,3-dioxygenase (IDO); and (3) the serotonin (5-hydroxytryptamine (5-HT)) production pathway in enterochromaffin cells via Trp hydroxylase 1. We focus on the first pathway because this is an article about gut microbes. The dominant products are indole and its derivatives. Indole, as an interspecies and interkingdom signaling molecule, plays important roles in bacterial pathogenesis and eukaryotic immunity, and indole concentrations of up to 1.1 mM are produced by indole-producing bacteria in the mouse, rat, and human gut [53, 54]. Most indole derivatives are considered as ligands for AhR (aryl hydrocarbon receptor) such as indole-3-acetaldehyde (IAAld) and indole-3-aldehyde (IAld). The AhR recognizes xenobiotics as well as natural compounds such as dietary components and microbiota-derived factors. AhR affects T  cell differentiation and Th17 development and upregulates the IL-22 level to maintain the immune homeostasis in the intestinal tract [51, 55]. This is consistent with that there are lots of ligands existing in human gut such as indole as well as its derivatives. Some scientists find that highly adaptive *lactobacilli* are expanded and produce an AhR ligand (indole-3-aldehyde) that contributes to AhR-dependent IL-22 producing. The resulting phenomenon also provides antifungal resistance to fungus *Candida albicans* [56]. The uncovered mechanism provides us a new sight into interactions among host, indigenous bacteria, and harmful foreign pathogens. Other Trp-derived indole derivatives such as I3S (indoxyl-3-sulfate) reduced *Ccl2* and *Nos2* expressions in astrocytes in an AhR-dependent manner [57]. The article also reports that the AhR expression could be enhanced by the IFN-I signaling in astrocytes with upregulation of genes associated with IFN-I signaling. These findings suggest that it is promising to limit the central nervous system (CNS) inflammation by combing drugs and diets therapy because of the important roles of astrocytes during CNS injury and disease. Another derivative, indole 3-propionic acid (IPA), is proved as an agonist for the pregnane X receptor (PXR) and downregulated the enterocyte inflammatory cytokine tumor necrosis factor-*α* (TNF-*α*) via toll-like receptor 4 (TLR4) [58]. In general, tryptophan derivatives are almost harmless to humans and may have an organ-specific or species-specific interaction with the host.

### 2.6. Phenylalanine Metabolites

The phenylalanine absorbed by the host is either utilized by the host or intestinal microbiota. Mostly, diet phenylalanine is digested into tyrosine with the help of phenylalanine hydroxylase (PAH) and then involved in melanin metabolism. The left is converted to phenylpyruvic and phenylacetic with the help of phenylalanine ammonia lyase (PAL). The patients with phenylketonuria caused by the accumulation of toxic metabolites of phenylalanine have interferences in these two enzymes. Some researchers [59] characterize a pathway from the gut symbiont *Clostridium sporogenes* that generates phenylalanine acid metabolites. This species either metabolizes phenylalanine to corresponding propionic acid derivatives phenylpropionic acid (PPA) with the enzymes encoded by *fldH, fldBC*, and *acdA* or phenylacetic acid (PAA) with the enzyme encoded by *porA*. A recent study [60] shows that PAA serves as the precursor of the gut microbiota-generated metabolite phenylacetylglutamine (PAGln), and phenylacetylglycine (PAGly) would promote cardiovascular disease- (CVD-) relevant phenotypes via host G protein-coupled receptors (GPCRs), including a2A, a2B, and b2-adrenergic receptors (ADRs). It is also worth mentioning that the production of PAGln and PAGly is species-specific, which means the PAA could be the precursor of different phenylalanine derivatives in different biological intestines with different microflora. All these results indicate that what matters are the enzymes that the microbes have, not the microbes themselves. Thus, it will be interesting to create an engineering bacterium which can turn toxic metabolites of phenylalanine into beneficial metabolites with its special enzyme ratio. In this way, people could prevent some diseases induced by losing certain enzyme such as phenylketonuria and enjoy food without any menace from the “rear.”

## 3. Conclusion

The gut microbiome has attracted increasing attention over the last 15 years. However, the abundance of metagenomic data generated on comparing diseased and healthy subjects can lead to the erroneous claim that a bacterium is causally linked with the protection or the onset of a disease. In fact, during the development of diseases, people are constantly changing their eating habits. Thus, the gut microbiome is changing too. We still need more work to go beyond the simple associations, and we need to provide as much as possible more complex analyses (e.g., multiomics and time series measurements) if we want to finally approach the final causality. For example, *P. copri* is found having the opposite effect in diabetes [13, 61]. This is consistent with that there are lots of research studies indicating that gut microbiota brings damage or benefits to host. There are lots of confounding factors that affect the specific role of gut microbiota. Thus, the targeted screening of gut microbiota could be realized by the host through diet control or fecal microbiota transplantation.

## Data Availability

No data were used to support this study.
